# Supplementary material for: Human Keratinocytes Inhibit CD4+ T-Cell Proliferation through TGFB1 Secretion and Surface Expression of HLA-G1 and PD-L1 Immune Checkpoints
Source: Cells. 2021 Jun 8;10(6):1438. doi: 10.3390/cells10061438 (PMC8227977; doi:10.3390/cells10061438)
Supplement: Supplementary file 1 [file cells-10-01438-s001.zip › cells-1236076-supplementary.pdf]

cells

MDPI

Article

Human Keratinocytes Inhibit CD4+ T-Cell Proliferation through TGFB1 Secretion and Surface Expression of HLA-G1 and PD-L1 Immune Checkpoints

Guillaume Mestrallet [1,2], Frédéric Auvré [1,2], Chantal Schenowitz [3,4], Edgardo D. Carosella [3,4], Joel LeMaoult [3,4,*,†], Michèle T. Martin [1,2,*,†], Nathalie Rouas-Freiss [3,4,*,†] and Nicolas O. Fortunel [1,2,*,†]

1 CEA, Laboratory of Genomics and Radiobiology of Keratinopoiesis, Institute of Cellular and Molecular Radiobiology, Francois Jacob Institute of Biology, DRF, 91000 Evry, France; guillaume.mestrallet@cea.fr (G.M.); frederic.auvre@cea.fr (F.A.)
2 Université Paris-Saclay, 91190 Saint-Aubin, France
3 CEA, DRF, Francois Jacob Institute of Biology, Hemato-Immunology Research Department, Saint-Louis Hospital, 75010 Paris, France; chantal.schenowitz@cea.fr (C.S.); edgardo.carosella@cea.fr (E.D.C.)
4 U976 HIPI Unit, IRSL, Université Paris, 75010 Paris, France
* Correspondence: joel.lemaoult@cea.fr (J.L.); michele.martin@cea.fr (M.T.M.); nathalie.rouas-freiss@cea.fr (N.R.-F.); nicolas.fortunel@cea.fr (N.O.F.); +33-1-60-87-34-91 (M.T.M.); +33-1-57-27-68-01 (N.R.-F.); +33-1-60-87-34-92 (N.O.F.)
† These authors contributed equally to this article.

Supplementary Materials

Figure S1. Maps of HLA-G1 and control vectors.

Figure S2. Fibroblast supernatant and PBMC proliferation inhibition.

Figure S3. HLA-G isoform expressed in keratinocytes.

Figure S4. Influence of EGF on PD-L1 expression in keratinocytes.

Figure S5. Impact of induced HLA-G1 overexpression on PD-L1 and TGFB1 expression in keratinocytes.

Figure S6. Cell-surface expression of the HLA-G receptors ILT2 and ILT4 in keratinocytes.

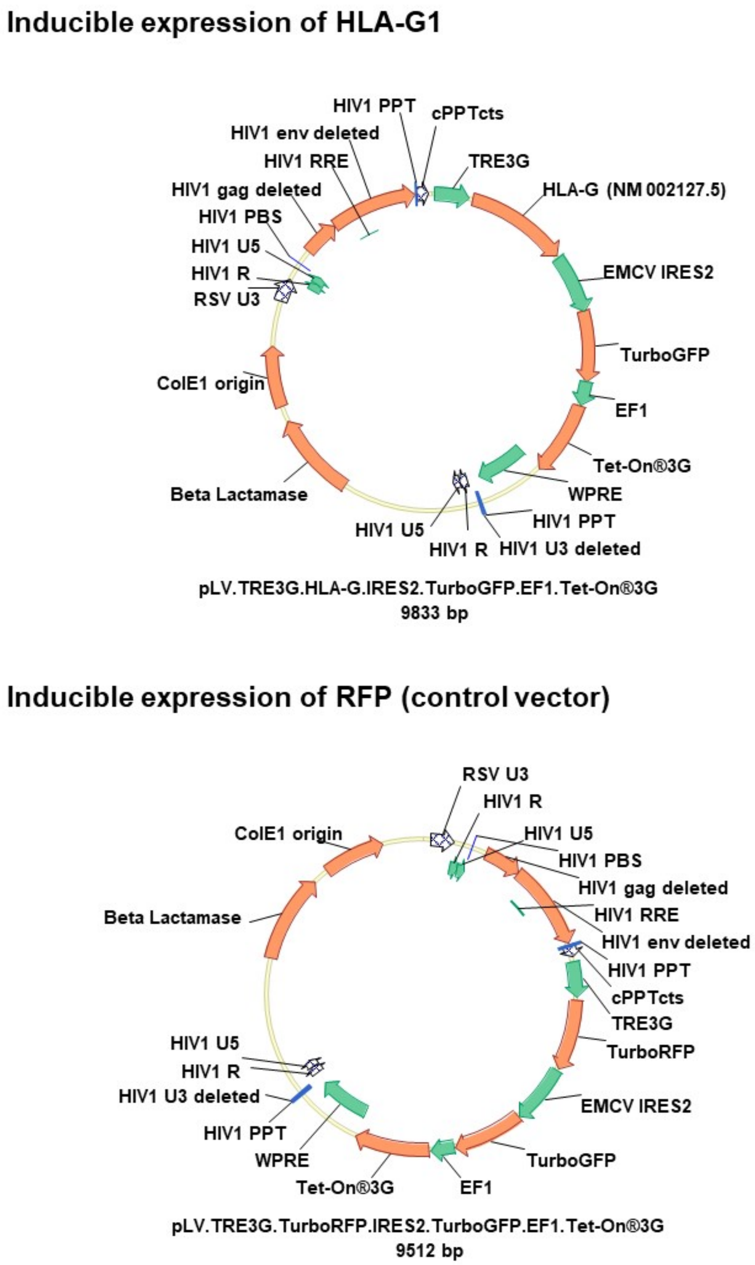

Figure S1. Maps of HLA-G1 and control lentiviral vectors.

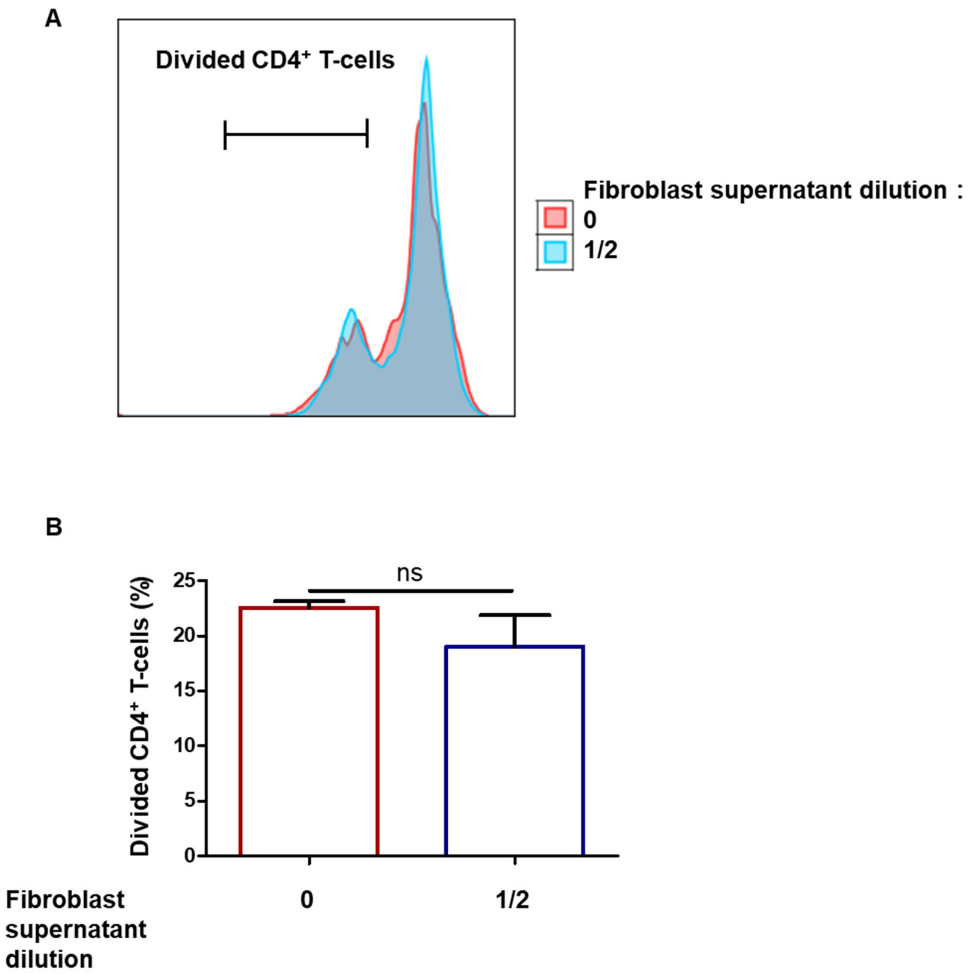

Figure S2. Fibroblast supernatant and PBMC proliferation inhibition. Fibroblast supernatants from one representative donor were incubated with 100,000 PBMCs for 7 days. PBMCs were pre-marked with a dye and activated using $CD3^{+}$ $CD28^{+}$ beads. PBMC proliferation was quantified by dye decrease at day 7. (**A**) Representative flow cytometry profiles at day 7. (**B**) $CD4^{+}$ T-cell proliferation depending on presence of fibroblast supernatant (mean+SEM, $p < 0.05$, $n = 3$). Exact p-values were determined on t-test.

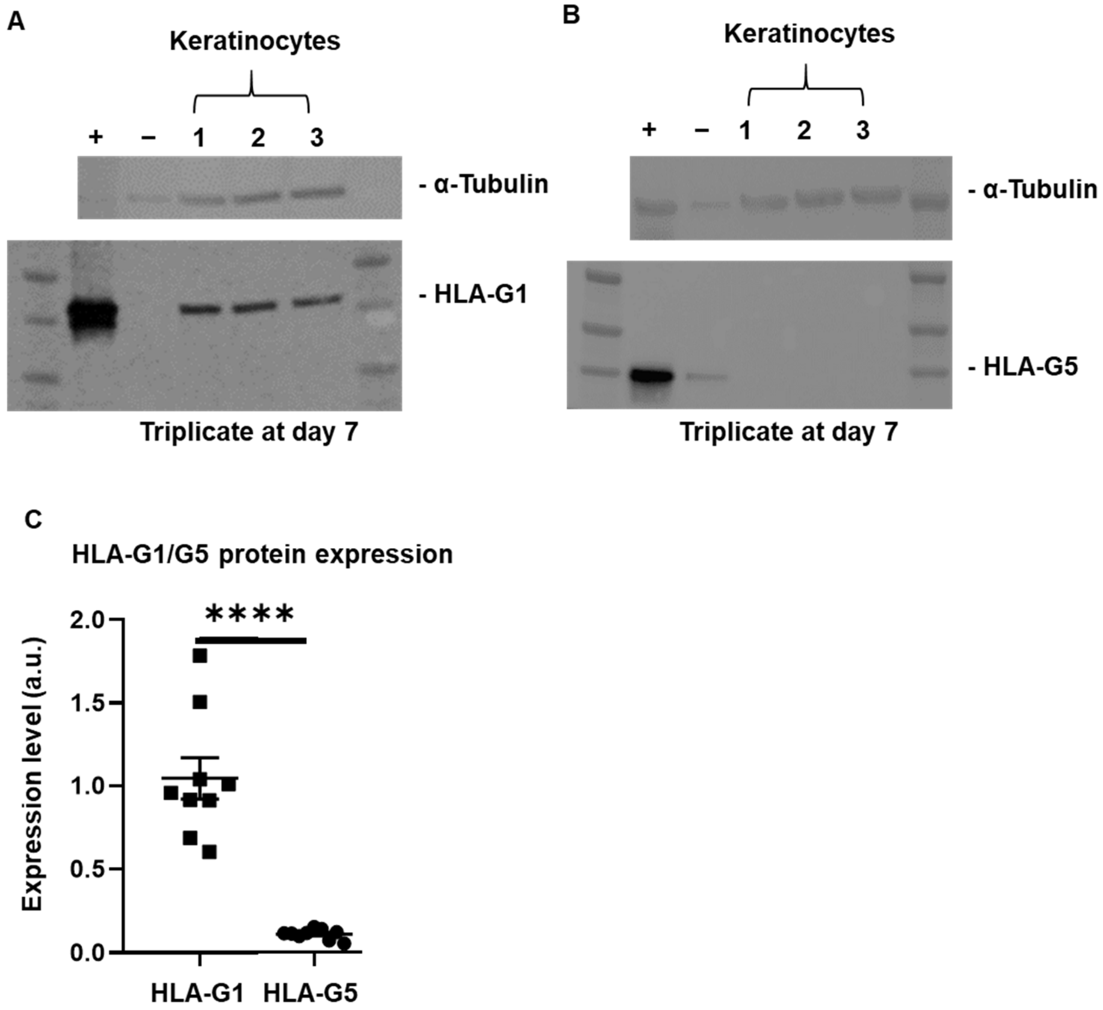

Figure S3. HLA-G isoform expressed in keratinocytes. Cells from one representative donor were cultivated for 7 days after removal in an undefined medium with serum and a layer of feeder cells. Analysis by Western blotting. (**A**) Typical gel photograph corresponding to 3 different cultures, with α-tubulin detection as loading control. K562 cells were used as negative control and K562 cells transduced with HLA-G1 were used as positive control with 4H84 antibody. (**B**) Typical gel photograph corresponding to 3 different cultures, with α-tubulin detection as loading control. M8 cells were used as negative control and M8 cells transduced with HLA-G5 were used as positive control with 5A6G7 antibody. (**C**) Scatter plot of quantification (mean+SEM, $p < 0.0001$, $n = 9$). Exact p-values were determined using the Mann-Whitney U-test. ****: $p<0.0001$.

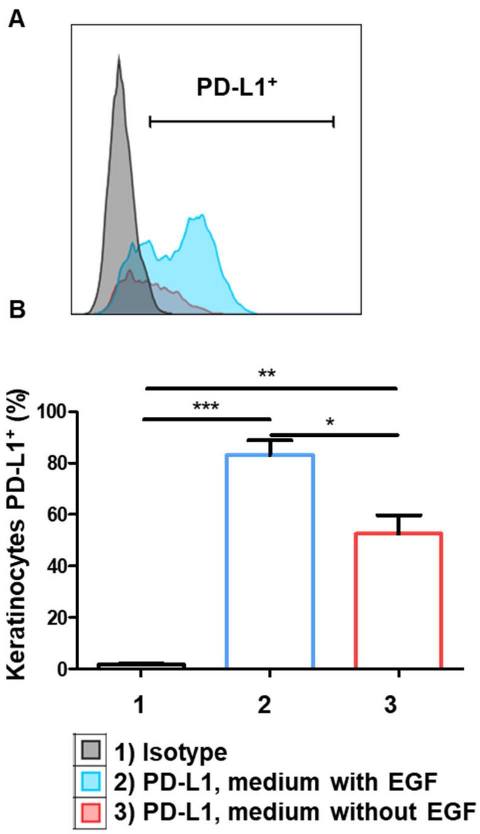

Figure S4. Influence of EGF on PD-L1 expression in keratinocytes. Amplified keratinocytes expressing PD-L1 were reamplified with medium with or without EGF. (**A**) Representative cytometry profiles after 7 days. (**B**) PD-L1 expression according to presence of EGF in the medium (mean+SEM, p < 0,05, n=3). Exact p-values were determined on t-test. *: p < 0.05, **: p < 0.01, and ***: p <0.001.

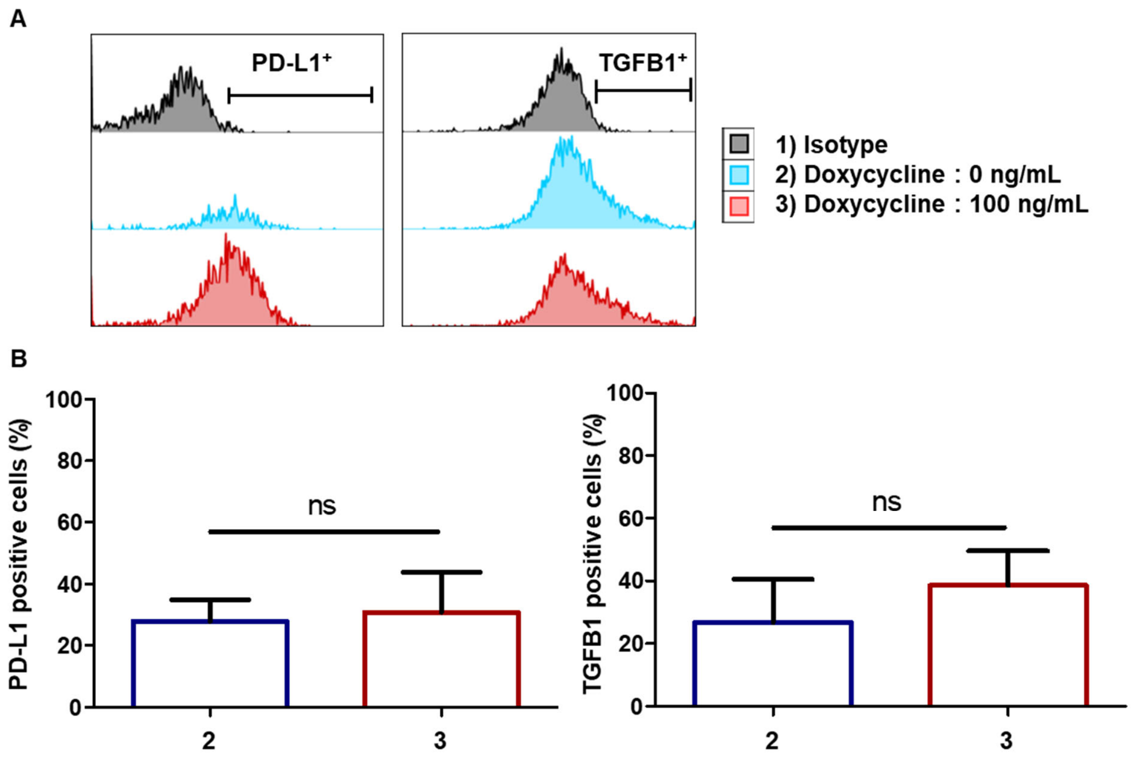

Figure S5. Impact of induced HLA-G1 overexpression on PD-L1 and TGFB1 expression in keratinocytes. Keratinocytes cultures of transduced with the lentiviral vector allowing doxycycline-inducible expression of HLA-G1 were maintained for 7 days in induced and non-induced conditions. Expression of PD-L1 and TGFB1 proteins was analyzed in both control and HLA-G1-overexpressing keratinocytes. (**A**) Representative profiles of PD-L1 and TGFB1 expression are shown. (**B**) Absence of PD-L1 and TGFB1 protein expression modulation in response to HLA-G1 overexpression induced by doxycycline treatment (mean+SEM, $p<0.05$, $n=3$). Exact p-values were determined using the t-test.

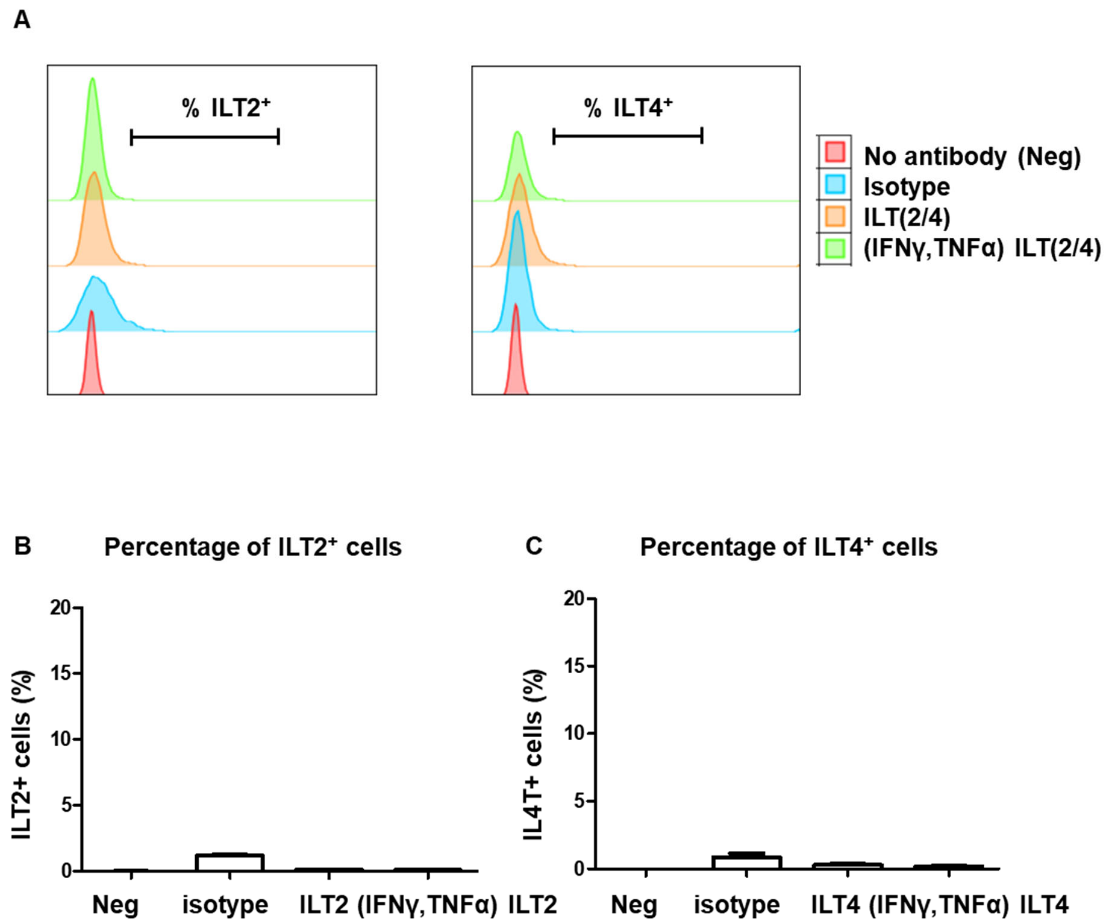

Figure S6. Cell-surface expression of the HLA-G receptors ILT2 and ILT4 in keratinocytes. Cell cultures were performed during 2 days in the absence or presence of 10 ng/mL of IFNγ and 10 ng/mL TNFα to mimic an inflammatory context, and keratinocytes were analyzed for ILT2 and ILT4 cell-surface expression by flow cytometry. (**A**) Representative profiles are shown. Absence keratinocytes positive for both ILT2 (**B**) and ILT4 (**C**) cell-surface expression.
